# Supplementary material for: HKG: an open genetic variant database of 205 Hong Kong cantonese exomes
Source: NAR Genom Bioinform. 2022 Feb 8;4(1):lqac005. doi: 10.1093/nargab/lqac005 (PMC8826781; doi:10.1093/nargab/lqac005)
Supplement: lqac005_Supplemental_Files [file lqac005_supplemental_files.zip › Supplementary_Note.hkg_nargb.resubmission2.draft_20211204.docx]

Supplementary Note of

HKG: An open genetic variant database of 205 Hong Kong Cantonese exomes

Min Ou, Henry Chi-Ming Leung, Amy Wing-Sze Leung, Ho-Ming Luk, Bin Yan, Chi-Man Liu, Tony Ming-For Tong, Myth Tsz-Shun Mok, Wallace Ming-Yuen Ko, Wai-Chun Law, Tak-Wah Lam, Ivan Fai-Man Lo, Ruibang Luo

# IBD analysis using HKG and CHN exome data

## Method

For the IBD analysis, variants in HKG were phased using Beagle 4.1. The 1KGP phase 3 variants (1KGPp3 20170504) were used as the reference panel. Only the targeted exome positions were included in the IBD analysis. All HKG and 1KGP CHN variants were processed by the Beagle v4.1 IBD calling algorithm with 15 iterations, each time using a different random seed. In each iteration the minimum length of the IBD was set to 3 cM to minimize the influence of phasing and genotyping errors. We combined the results of the 15 iterations using the “ibdmerge” module (<https://faculty.washington.edu/browning/beagle_utilities/utilities.html#ibdmerge>). The normalization method from Nakatsuka et al. 2017 (1) was used to enable a population-wise comparison.

For the IBD detection using HKG samples, we further improved the IBD detection confidence by considering the relationship between the detected IBD segment and the sequenced regions. We filtered out those IBD segments if over 80% of the sequenced regions in a segment are on the left or right 10% boundary. In addition, 10% of the detected IBD segments with the lowest sequenced region coverage were removed.

## Result

Identical-by-Descent (IBD) analysis using the phased variants of CHN and HKG to estimate the effective population size and understand the population demography (2). We analyzed 593 phased samples (CHB: 108; CDX: 109; CHS: 171; HKG: 205) and detected 2,585 filtered IBD segments. Supplementary Table S2 summarizes the number of detected IBD segments, their total length, and the corresponding number of individuals in each population after all filters. CDX had the highest normalized IBD shared and was 10 times higher than that in CHB. A smaller normalized length of IBD in CHB might be due to its large effective population size or diverse sampling. The limited effective population size detected in CDX also agreed with the fact that CDX was a geographically isolated population (in Xishuangbanna of Yunnan province in southwestern China). Having a similar normalized per-segment length, HKG has a level of genetic relatedness like CHS. This suggests that the HKG is not as isolated as the CDX and has a lower population mixture than the CHB.

## Reference

1. Nakatsuka, N., Moorjani, P., Rai, N., Sarkar, B., Tandon, A., Patterson, N., Bhavani, G.S., Girisha, K.M., Mustak, M.S., Srinivasan, S., et al. The promise of discovering population-specific disease-associated genes in South Asia. *Nat. Genet.* 2017; 49:1403-1407

2. Nait Saada, J., Kalantzis, G., Shyr, D., Cooper, F., Robinson, M., Gusev, A. and Palamara, P.F. Identity-by-descent detection across 487,409 British samples reveals fine scale population structure and ultra-rare variant associations. *Nat. Commun.* 2020; 11:6130
